# Transmembrane-4 L-six family member-1 (TM4SF1) promotes non-small cell lung cancer proliferation, invasion and chemo-resistance through regulating the DDR1/Akt/ERK-mTOR axis

**DOI:** 10.1186/s12931-019-1071-5

**Published:** 2019-05-29

**Authors:** Lin Ye, Chunyun Pu, Jun Tang, Yan Wang, Can Wang, Zhu Qiu, Tingxiu Xiang, Yunmei Zhang, Weiyan Peng

**Affiliations:** 1 Department of Cardiothoracic Surgery, The First Affiliated Hospital of Chongqing Medical University, Chongqing, China; 2 Chongqing Key Laboratory of Molecular Oncology and Epigenetics, The First Affiliated Hospital of Chongqing Medical University, Chongqing, China; 3 Department of Oncology, Chongqing University Cancer Hospital& Chongqing Cancer Institute, Chongqing, China; 4 Nursing College, Chongqing Medical University, Chongqing, China

**Keywords:** Non-small-cell lung cancer, TM4SF1, Invasion, Migration, Apoptosis, Cell cycle, Chemo-sensitivity, DDR1, Akt, ERK

## Abstract

**Background:**

Tumor chemo-resistance is a hallmark of malignant tumors as well as the major cause of poor survival rates in lung cancer. *Transmembrane-4 L-six family member-1 (TM4SF1)*, an antigen that serves as an oncogene, mainly affects tumor invasion and metastasis. We investigated the roles of *TM4SF1* in non-small-cell lung cancer progression, particularly in the regulation of chemo-sensitivity.

**Methods:**

*TM4SF1* was silenced by small interfering RNA transfection.*TM4SF1* expression in cell lines and tissues were determined by Quantitative Real-time PCR. MTS, clonogenic, Transwell assay, Flow cytometry verified cell function. By RT-PCR, Western blot, the mechanisms were studied.

**Results:**

*TM4SF1* was upregulated in both lung cancer cell lines and tissues, compared with 293 T epithelial cells. Analysis of online databases revealed that high expression of *TM4SF1* is associated with the older patient age, smoking habits, and poor patient survival and outcome. Knockdown of *TM4SF1* substantially inhibited tumor cell growth, migration, and invasion, and enhanced the chemo-sensitivity of the lung cancer cell lines A549 and H1299 to cisplatin and paclitaxel. Furthermore, the silencing of *TM4SF1* induced lung cancer cell apoptosis and arrested cells at the G2/M phase. These results suggest that *TM4SF1* is associated with lung cancer progression and appears to be required for tumor cell growth, maintenance of chemo-resistance and metastasis. We further found that TM4SF1 exerts these effects in part by regulating the expression of the discoidin domain receptor DDR1 and its downstream target, the Akt/ERK/mTOR pathway, and consequently alters cell sensitivity to chemo-reagents and contributes to invasion and metastasis.

**Conclusions:**

These findings demonstrate that TM4SF1 may serve as a prognostic factor for lung cancer chemo-response and patient outcome.

## Background

Lung cancer is the most common cancer worldwide, with an incidence of 11.6% [1]. Non-small-cell lung cancer (NSCLC) constitutes 85 to 90% of lung cancers and is the most common cause of cancer death [2]. Cisplatin- or paclitaxel-based adjuvant therapy is one of the major treatment approaches among the systemic therapies for NSCLC. This method has been demonstrated to have unequivocal benefits, in terms of both increasing cure rates in adjuvant and multimodality settings in earlier stages of the disease and enhancing the quality and length of life in advanced stages of the disease [2]. However, data from more than 1300 patients enrolled in eight trials of adjuvant cisplatin-based therapy have indicated a 5% survival benefit at 5 years. These disappointing results are partially due to the chemo-resistance of NSCLC [3]. Therefore, understanding the mechanisms of how NSCLC becomes resistant to chemo-reagents is crucial.

*Transmembrane-4 L-six family member-1 (TM4SF1)* is a small plasma membrane glycoprotein that regulates cell motility and proliferation [4]. *TM4SF1* was first discovered as an antigen for immunotherapy in lung cancer and its antibody MAb L6 showed a favorable binding properties and the encouraging clinical effect [5]. Experiments on tumor cells have previously shown *TM4SF1* to be important for cell growth in liver and lung cancer [6, 7], motility in lung cancer [7], invasion in pancreatic cancer [8], and metastasis of breast cancer to the lungs [9]. *TM4SF1* has been reported to interact with *discoidin domain receptor 1 (DDR1)* in breast cancer [9] and in pancreatic cancer metastasis [10]. *DDR1* is an up-stream regulator of the *AKT/mTOR* pathway [11], a pathway involved in chemo-resistance in multiple cancers, including lung cancer [12]. Consequently, we hypothesized that *TM4SF1* might also participate in the process of cancer chemo-resistance through regulating *DDR1*. However, the role of *TM4SF1* in lung cancer chemo-sensitivity has not been investigated. We report here that *TM4SF1* regulates lung cancer chemo-sensitivity and apoptosis through the *DDR1*-activated *AKT/mTOR* signaling pathway.

## Methods

### Cell lines and tumor samples

NSCLC cell lines (A549, H1299) were purchased from the ATCC (Manassas, VA,USA). Both cell lines are lung adenocarcinoma cell lines. Cell lines were cultured in RPMI 1640 as described previously [13]. Human embryonic kidney epithelial cell 293 T cell lines were used as a normal control. 293 T cells were cultured in DMEM with 10% Fetal Bovine Serum. Fresh NSCLC tissues and tumor-adjacent tissues were obtained from patients who underwent lobectomy at the Department of Cardiothoracic Surgery (Patient clinical features were listed in Table 1) in the First Affiliated Hospital of Chongqing Medical University (Chongqing, China). This research was approved by the Institutional Ethics Committees of the First Affiliated Hospital of Chongqing Medical University and followed the principles of the Declaration of Helsinki. Patient consent forms were signed by each patient who participated in this study.

**Table 1:** Clinicopathological features of 25 NSCLC patients

Clinicopathological features	Number(*n* = 25
Gender	
Male	20
Female	5
Age	
≤ 50	5
> 50 < 60	17
>60	3
Phase	
I	9
II	6
III	7
IV	3
Tumour size	
< 3.0 cm	9
≥ 3.0 cm ≤ 5.0 cm	13
≥ 5.0 cm ≤ 7.0 cm	2
≥ 7.0 cm	1
Lymph node metastasis	
present	8
absent	17
Distant Metastasis	
present	4
absent	21
Smoking status	
smoker	17
non-smoker	8
Tumor Histology	
Adenocarcinoma	11
SqCa	13
Adeno+SqCa	1

### RNA extraction

RNA was extracted from cell lines and tissue samples with TRIzol reagent (Molecular Research Center, Cincinnati, OH, USA) and treated with DNase I. The RNA was stored at − 80 °C, and its concentration was measured by spectrophotometry. RNA was reverse-transcribed according to the instructions of the Reverse Transcription System (Promega, Madison, WI, USA). Semi-quantitative PCR was carried out with Go-Taq DNA polymerase (Promega), and reaction conditions were as previously described [12]. Real-time quantitative PCR used ABI SYBR Green on an ABI 7500 Real-Time PCR Detection System (Applied Biosystems, Foster City, CA, USA). GAPDH was used as a loading control.

The primers designed for human TM4SF1 were as follows: forward, 5′-GGTTCTTTTCTGGCATCGTAGGAGGTG-3′;reverse,5′-CTGGCCGAGGGAATCAAGACATAGTG-3′. The primer for GAPDH was as follows: forward,5′-GGAGTCAACGGATTTGGT-3′; reverse,5′-GTGATGGGATTTCCATTGAT-3′.

### Knockdown of TM4SF1 with small interfering RNAs (siRNAs)

TM4SF1-target-specific siRNA, negative-control TM4SF1-target-specific siRNA and negative-control siRNA (NC) were all synthesized by OriGene, (Rockville, MD,USA), Three pairs of siRNAs that targeted TM4SF1 were designed and synthesized: siRNA-TM4SF1-A: 5′- GCGAUGCUUUCUUCUGUAUTT-3′ (forward), 5′-AUACAGAAGAAAGCAUCGCTT-3′(reverse); siRNA-TM4SF1-B:5′-GGCUCUUGGUGGAAUUGAATT-3′(forward), 5′-UUCAAUUCCACCAAGAGCCTT-31 (reverse); siRNA-TM4SF1-C: 5′-GCUCUCACCAACAGCAAUATT-3′(forward),5′-UAUUGCUGUUGGUGAGAGCTT-3′(reverse). The sequence of NC-siRNA was as follows: 5′-UUCUCCGAACGUGUCACGUTT-3′ (forward); 5′-ACGUGACACGUUCGGAGAATT-3′ (reverse). Transfections were performed using Lipofectamine-2000 transfection system as described previously [8].

### Colony-formation assays

A549 and H1299 cells transfected with siRNA or NC were plated in six-well plates at a density of 1200 cells/well in culture medium 24 h after transfection medium was changed every 3 daysAfter 14 d, the cells were fixed with 4% PFA and stained with crystal violet solution. Colonies with > 50 cells per colony were counted.

### Cell proliferation assays

Cell proliferation was assessed with MTS assays (Abcam, Cambridge, MA,USA) and PCNA (Proliferating Cell Nuclear Antigen) expression. PCNA is known as a marker of cell proliferation besides Ki67, testing PCNA expression could further understand the effects of TM4SF1 on NSCLC cell proliferation. For MTS, NC- and siRNA-transfected cells were seeded in 96-well plates (3000 cells/well) with 100 μL of medium. Cells were incubated for 24, 48 or 72 h, and then 100 μL/well serum-free medium containing 20 μL MTS reagent was added to each well. Plates were then incubated at 37 °C for an additional 2 h. Absorbance was measured at 490 nm with a microplate reader (Multiskan MK3; Thermo Fisher Scientific,). PCNA expression was carried out by western blot.

### Chemo-reagent treatment

Cisplatin and paclitaxel were purchased from QiLu Pharma (Shan Dong, China). TM4SF1 knockdown cells and NC cells were treated with cisplatin at concentrations of 0, 2.5, 5, 10, 15, or 20 μg/ml for 12 h, or with paclitaxel at concentrations of 0, 2.5, 5, 10, 20, or 40 ng/ml at 24 h and then collected for MTS analyses to quantify cell viability of TM4SF1 knockdown cells and NC cells.

### Cell migration and invasion assays

Cell migration and invasion ability were measured with Transwell chambers (8-μm pore size; Corning, NY, USA), as described previously [14]. In general, 4 × 10^4^ cells/well diluted in 100 μl serum-free medium was in the upper transwell filter inserts in 24-well plates. The lower chambers contained 800 μl medium containing 10% FBS. Cells were incubated at 37 °C in a 5% CO_2_ incubator for 24 h. For invasion assay, the upper chamber was pre-coated with 1:5 diluted Matrigel™.

### Flow cytometry analysis of the cell cycle and apoptosis

For apoptosis, 1 × 10^6^A549 and H1299 cells were transiently transfected with siRNA or NC, follow the manufacture’s instruction of Lipofectamine-2000 transfection system, then analyzed with Annexin V-FITC/PI(5ul/1 × 10^6^cell) following the manufacture’s protocol (BD Medical Technology) staining for apoptosis analysis. Data were analyzed with CellQuest™ Pro (BD Biosciences, San Jose, CA, USA).

For cell cycle analysis, cells were collected as before. Cells were then sorted with a FACSCalibur machine (BD Biosciences, Franklin Lakes, NJ, USA). Data were analyzed in ModFit 3.0 software (Verity Software House, Topsham, ME, USA).

### Western blotting

SiRNA- *TM4SF1*or NC-transfected cells (1 × 10^6^) were washed three times with ice-cold PBS and then lysed in lysis buffer (4%v SDS, 20% glycerol,120 mM Tris-HCI, PH 6.8). Then, 40 μg protein samples were separated by SDS–PAGE, transferred to polyvinylidene difluoride membranes and immune-stained as previously described [15]. Signals were detected with an ECL Plus Western Blotting Detection Reagents (RPN2132, GE HealthcareLife Sciences, UK). Primary antibodies to the following targets were used: TM4SF1 (sc-103,267) and p-ERK (sc-7383), both Santa Cruz Biotechnology, CA, USA, cleaved caspase-3 (#9661), cleaved caspase-7 (#9491, cleaved caspase-9 (#9504), cleaved PARP (#9661), DDR1 (#3917), p-mTOR (#5536, Cell Signaling, MA), and p-AKT (#4060), all Cell Signaling Technology, Danvers, MA, USA, with GAPDH (bsm-51,010 M; BIOSS, China), as a loading control. The dilution of primary and secondary antibodies was used according to the company’s recommendations, Primary antibodies were diluted in 1:1000, secondary antibodies were diluted in 1:3000. The bands were detected with an enhanced chemiluminescence kit (Amersham Pharmacia Biotech, Piscataway, NJ, USA).

### mTOR inhibitor treatment

TM4SF1 knocking down H1299 cell lines and NC cells were treated with mTOR inhibitor Dactolisib at 50 μM for 24 h [16]. Protein was then extracted and western blotting was used as described before.

### Analyses using online databases

The Human Protein Atlas (https://www.proteinatlas.org/) were used to analyze the correlation between *TM4SF1* expression and patient survival in NSCLC. The relationships between *TM4SF1* expression and NSCLC patient clinical signatures were analyzed with the UALCAN database (ualcan.path.uab.edu/). The threshold search value used for this study was a *p*-value < 0.05.

### Statistical analysis

All data are representative of three independent experiments and are presented as mean ± SD. SPSS16.0 software was used for statistical analyses. For all tests, *p* < 0.05 was considered statistically significant.

## Results

### *TM4SF1* is over-expressed in lung cancer cell lines and lung cancer tissue samples

**Fig. 1:**
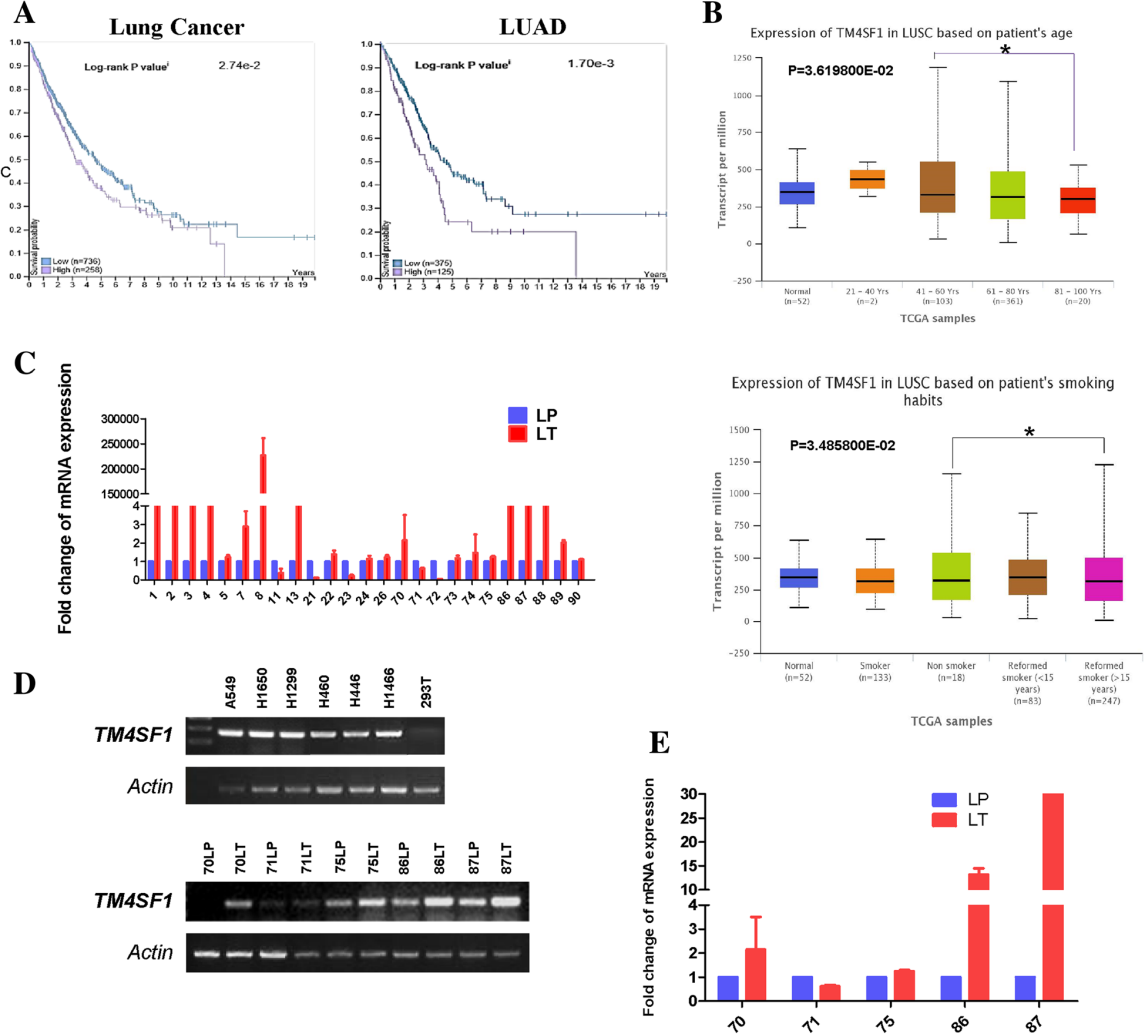
*Over-expression of TM4SF1 in NSCLC and it was associated with patients’ pathological features.* **a** The high expression of *TM4SF1* expression in NSCLC was associated with poor patient survival, data from The Human Protein Atlas (https://www.proteinatlas.org/). **b** The high expression of TM4SF1 was related to patient age and smoking habits,data from UALCAN database (ualcan.path.uab.edu/).**c** The expression of *TM4SF1* in NSCLC tissues and non-tumor adjacent surgical margine by q-PCR. **d** RT-PCR demonstrated *TM4SF1* expression in NSCLC cell lines and tissue samples**. e** q-PCR verified the RT-PCR results of *TM4SF1* expression in RT-PCR

We sought to understand the biological function of *TM4SF1* in lung cancer, specifically whether it promotes or suppresses lung cancer development. We first evaluated its expression in both lung cancer cell lines and tissue samples. With RT-PCR, we identified that *TM4SF1* was up-regulated in the lung cancer cell lines A549, H1299, H1650, H460, H446, and H1466, compared with the epithelial cell 293 T cells (Fig. 1d upper panel), suggesting that it may potentially promote lung cancer. Furthermore, through real-time quantitative PCR, we quantified *TM4SF1* expression in 25 paired lung cancer tissue and its related adjacent non-tumor tissues. *TM4SF1* was over-expressed in the lung cancer tissues relative to the adjacent non-tumor tissues in 21 out of the 25 pairs (84%), suggesting that it may be an oncogene in lung cancer (Fig. 1c,e). RT-PCR was further used to confirm the *TM4SF1* expression in five pairs of lung tumor tissues and non-tumor tissues (Fig. 1d lower panel). These findings together demonstrated that *TM4SF1* is up-regulated in both lung cancer cell lines and tumor tissues, and it may be a potentially related to lung cancer progression.

### *TM4SF1* expression is related to patient clinicopathological features and outcomes

To assess any correlation between *TM4SF1* gene expression and patient clinicopathological features in NSCLC samples, we used the UALCAN database (www.ualcan.path.uab.edu/). The results showed that in lung squamous cell cancer, which accounts for most (80%) cases of lung cancer, high *TM4SF1* expression was associated with older patient age, in addition, its high expression was related to patient smoking status (Fig. 1b), with *TM4SF1* being expressed more highly in former smokers than in non-smokers .

We further analyzed the relationship between *TM4SF1* expression and NSCLC patient survival the Human Protein Atlas database (www.proteinatlas.org/ ). The Human Protein Atlas database confirmed in both lung adenocarcinoma (*p* = 0.0017) and lung cancers, including both squamous cell cancer and adenocarcinoma (*p =* 0.00274), high *TM4SF1* expression was associated with poor patient survival)(Fig. 1a). These results indicated that TM4SF1 expression may serve as an early biomarker to predict the prognosis of lung cancer patients.

Together, these findings indicate that high *TM4SF1* expression, an age over 80, a former smoking habit are risk factors for poor patient outcome in lung cancer. TM4SF1 may serve as a biomarker of lung cancer outcome and prognosis.

### Down-regulation of *TM4SF1* in A549 and H1299 cells suppresses cell growth, migration, and invasion

**Fig. 2:**
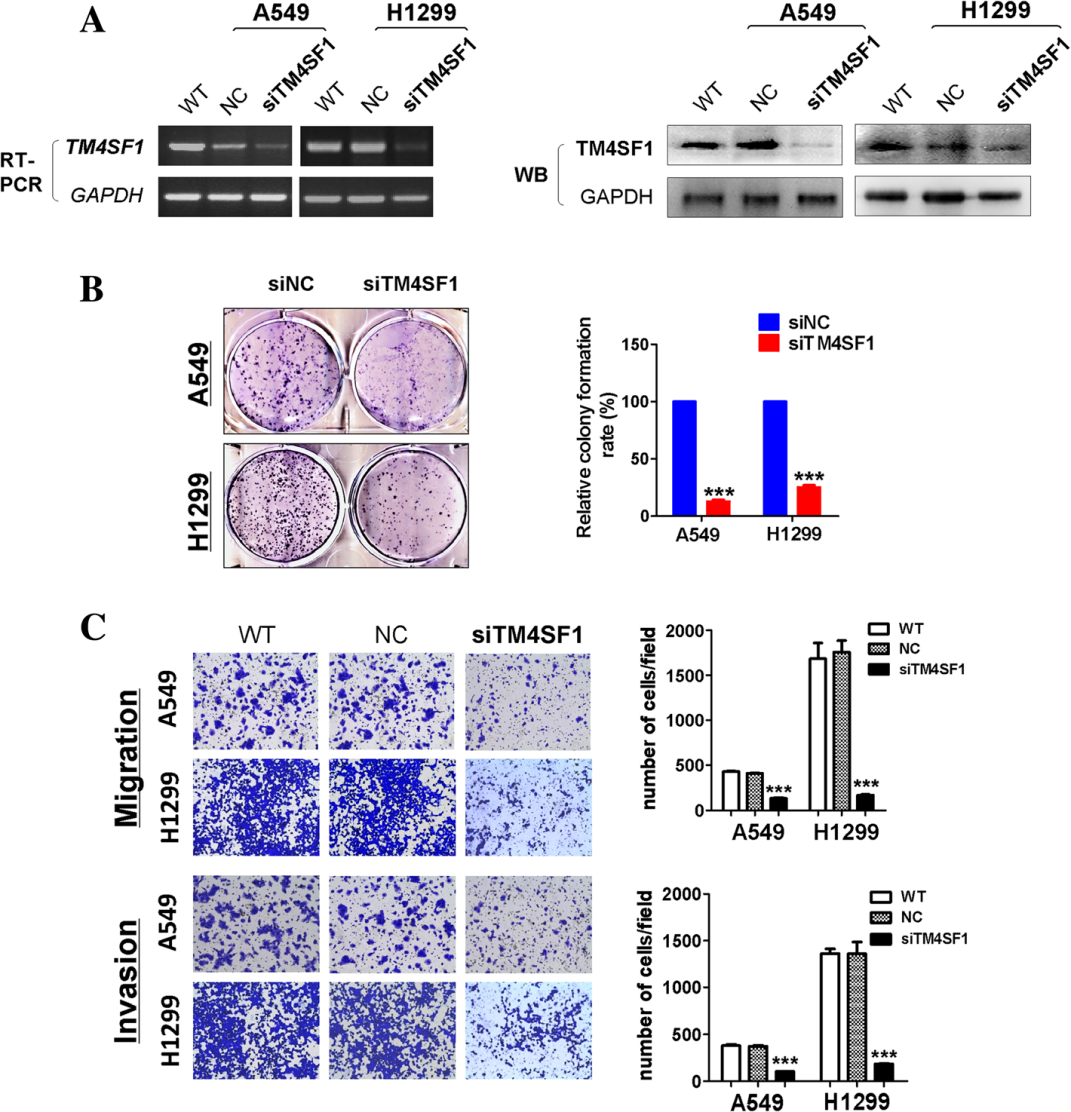
*The knockdown of TM4SF1 suppressed colony formation and cell invasion and migration in A549 and H1299 cell lines.* **a** RT-PCR and western blots confirmed the silencing of *TM4SF1* in A549 and H1299 cell lines. **b** The silencing of *TM4SF1* by siRNA inhibited colony formation in A549 and H1299 cell lines (left panel). Right panel showed quantitative analysis of colony formation. The numbers of colonies in NC cells were set to 100%. Values are expressed as the mean ± SD from three independent experiments, and the asterisk indicates the statistical significance compared to the controls (***, *p* < 0.001). **c** Cell migration and invasion assay in NC and TM4SF1 silenced A549 and H1299 cell lines by a 24-transwell system. Original magnification× 10, Asterisks indicate a significant level of proliferation compared with controls (***, *p* < 0.001), the right panel indicates the quantitative analysis of transwell cell invasion and migration assay

**Fig. 3:**
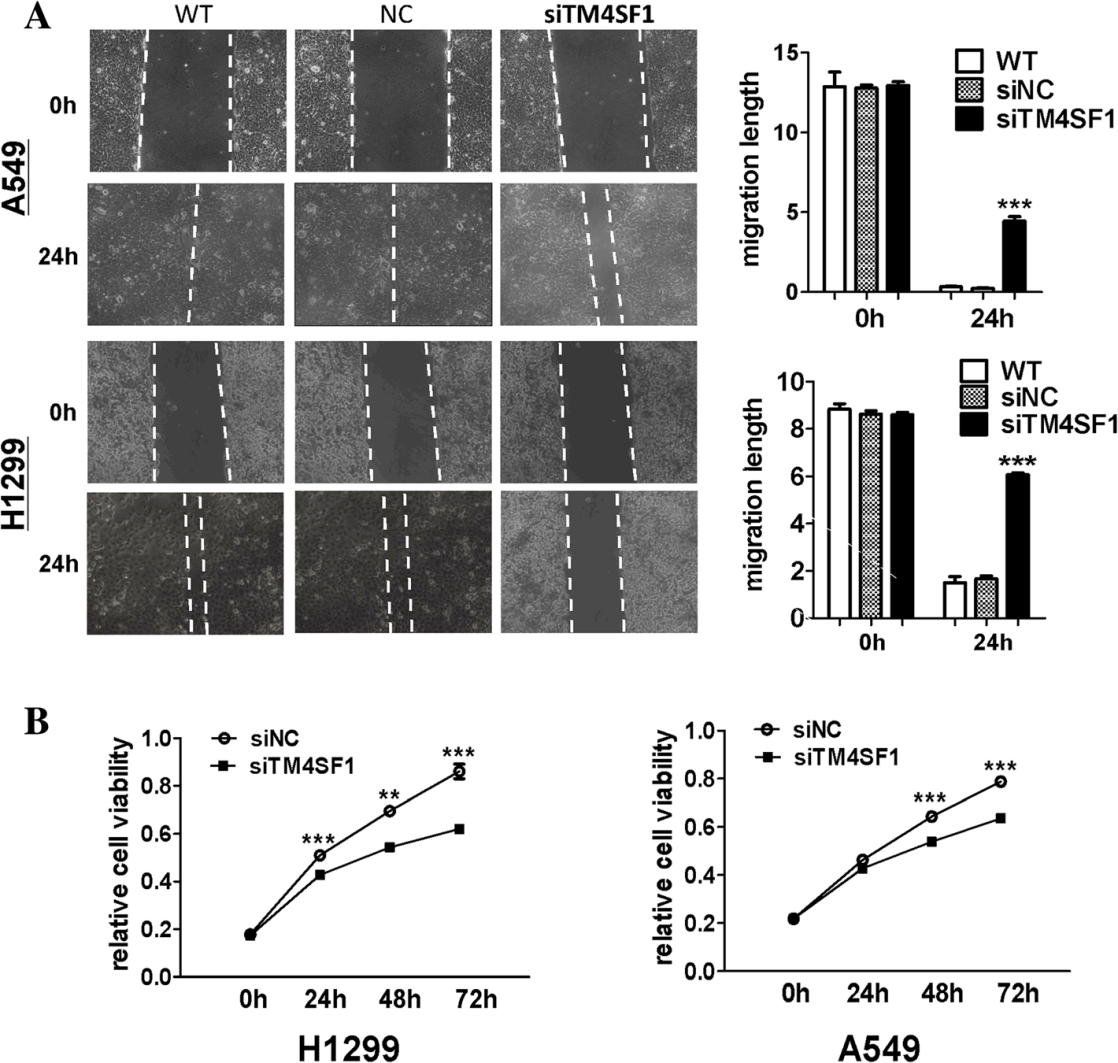
*The knockdown of TM4SF1 suppressed cell wound healing ability and cell proliferation in A549 and H1299 cell lines.* **a** Representative image (left) of wound healing assay in NC and TM4SF1 knocking down A549 and H1299 cells. The distance of cell migration was collected to make the statistical figure (right). **b** The ability of cell proliferation was measured in NC and TM4SF1 knocking cells by MTS. And the asterisk indicates the statistical significance compared to the controls (***, *p* < 0.001, **,*p* < 0.01)

**Fig. 4:**
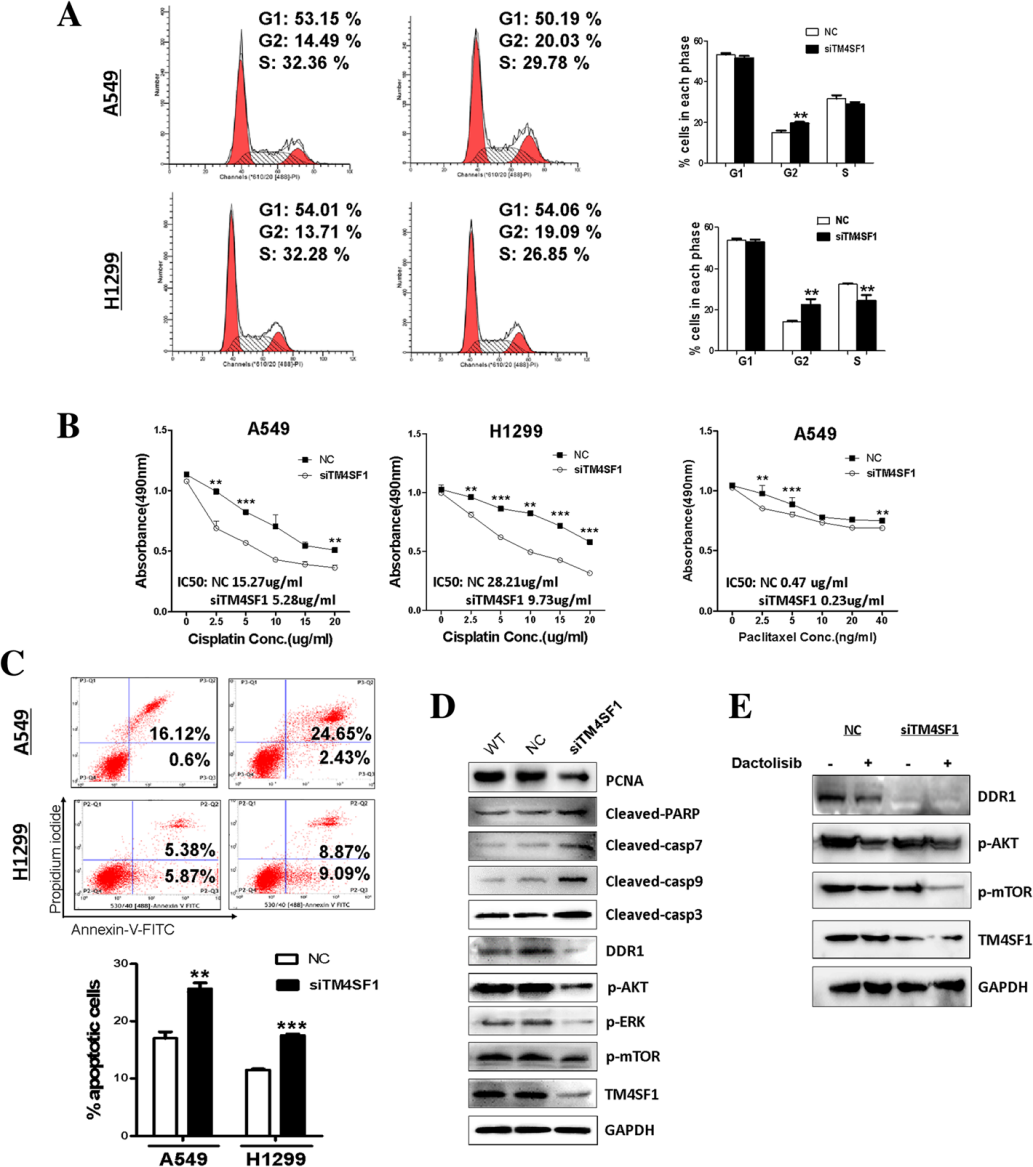
*The silencing of TM4SF1 induces cell cycle G2/M arrest, apoptosis and affects DDR1 signaling and apoptotic markers in NSCLC cell lines.* **a** Representative distribution of A549 and H1299 cell lines both in NC and TM4SF1 silencing cells (left); The distribution and percentage of cells in the G1, S and G2/M phases of the cell cycle are indicated (right).**b** Knocking down of TM4SF1 sensitized NSCLC cells to cisplatin and paclitaxel, The cell viability was detected by MTS assay after cells were treated with chemotherapy drugs paclitaxel for 24 h or cisplatin for 12 h. All experiments were performed in triplicate, respectively. *p < 0.05, **p < 0.01, ***p < 0.001. **c** Induction of apoptosis detected by flow cytometric analysis with Annexin V-FITC and PI-staining (right). Quantitative analysis of apoptosis (**p < 0.01, ***p < 0.001). **d** Expression levels of Casp7, 9, 3, cleaved-PARP were evaluated by western blot in NC and TM4SF1 silencing cells. And the impact of silencing TM4SF1 on the expression of DDR1 and the phosphorylation of its downstream targets AKT, ERK and mTOR. **e** Inhibitor down-regulated DDR1, mTOR and AKT and Knocking down TM4SF1 can further lower the expression of this pathway after inhibitor treatment

To investigate the function of *TM4SF1* in lung cancer cells, we transiently transfected A549 and H1299 cells with NC or siRNA-*TM4SF1* and confirmed the knockdown of *TM4SF1* by RT-PCR and western blotting. The results showed that both the *TM4SF1* mRNA and protein levels were significantly decreased by siRNA-*TM4SF1* (Fig. 2a). We then analyzed the effects of *TM4SF1* silencing on lung cancer cell proliferation, migration and invasion in vitro. By colony formation assay, we found the colonies formed in the *TM4SF1*-silenced group being smaller and fewer than those in the control group (*p* < 0.001) (Fig. 2 b). In Transwell chamber assays, silencing of *TM4SF1* markedly decreased the migration and invasion of lung cancer cells compared with wild-type and negative control cells (*p* < 0.001), (Fig. 4c). Moreover, wound healing assays revealed that scratches in *TM4SF1*-silenced A549 and H1299 cell lines healed significantly more slowly than those in negative control and wild-type cells 24 h after wounding (*p* < 0.001). The wound healing assays also indicated that knockdown of *TM4SF1* significantly inhibited lung cancer cell migration ability (Fig. 3a). With MTS, we analyzed the silencing of *TM4SF1* on cell viability and cell growth in lung cancer cell lines (Fig. 3b), by western blot,the PCNA expression was downregulated by knocking down *TM4SF1*(Fig.4 d) . Compared with the negative control, knockdown of *TM4SF1* significantly reduced cell viability (*p* < 0.001). Together, these findings suggested that *TM4SF1* inhibits lung cancer cell migration and invasion ability.

### *TM4SF1*-induced cell cycle arrest of NSCLC cells at the G2/M phase

Cell cycle regulation is one reason among the complex mechanisms of NSCLC chemo-resistance. To further test whether *TM4SF1* plays a role in cell cycle regulation, we performed flow cytometry cell cycle distribution assays. In the siRNA-*TM4SF1* transfected group, we found that the percentage of cells in the G2/M phase was greater than that in the control group (Fig. 4a), and the cell population in the S phase was also greater. These findings strongly suggested that the silencing of *TM4SF1* regulates cell cycle arrest at the G2/M phase.

### Silencing of *TM4SF1* enhances the chemo-sensitivity of NSCLC cells to paclitaxel and cisplatin

Because *TM4SF1* expression led to G2/M cell cycle arrest, we investigated the effect of *TM4SF1* knockdown on the chemo-sensitivity of NSCLC cells to cell cycle-phase-specific and phase-nonspecific chemotherapeutic drugs. Paclitaxel and cisplatin are the most commonly used chemo-therapy reagents for NSCLC. Paclitaxel functions by inducing cell G2/M arrest [14], while cisplatin is known as a cell cycle-phase-nonspecific chemo-reagent [12]. As shown in Fig. 4b, knocking down *TM4SF1* with siRNA increased the sensitivity of A549 and H1299 cells to paclitaxel and cisplatin, as compared with the controls. The IC50 of cisplatin in A549 /SiRNA-*TM4SF1* cells was 5.28 μg/ml vs 15.27 μg/ml in control cells; in H1299/SiRNA-*TM4SF1* cells, the IC50 was 9.73 μg/ml vs 28.21 μg/ml in NC H1299 cells (*p* < 0.001). In A549 cell line, the IC50 of paclitaxel was reduced by half by the silencing *of TM4SF1*. These results indicated that *TM4SF1* plays a role in the NSCLC chemo-resistance.

### *TM4SF1* induces apoptosis in NSCLC cells

The influence of *TM4SF1* on tumor cell proliferation suggested that it may induce cell apoptosis resistance in NSCLC cell lines. Flow cytometry of A549 and H1299 cells confirmed that, compared with control treatment, silencing of *TM4SF1* induced cell apoptosis in both cell lines (*p* < 0.01 in A549 cells and *p* < 0.001 in H1299 cells),(Fig. 4c). These results indicate that *TM4SF1* plays a part in NSCLC cell resistance to apoptosis and thus may be related to NSCLC chemo-resistance.

### *TM4SF1* enhances NSCLC chemo-sensitivity through induction of apoptosis and the DDR1/AKT signaling pathway

To further explore the mechanism underlying the ability of *TM4SF1* to induce NSCLC sensitivity to chemo-therapy, we used western blotting to verify the effects of *TM4SF1* on apoptotic markers and possible signaling pathways in NSCLC. *TM4SF1* knockdown significantly induced PARP (poly (ADP-ribose) polymerase) cleavage and up-regulated caspases 7, 9, and 3, suggesting that *TM1SF4* promotes lung cancer cell apoptosis and may induce lung cancer cell resistance to chemo-therapy-induced apoptosis, and play a part in lung cancer chemo-resistance. Furthermore, *DDR1* has been reported to interact with *TM4SF1* and to induce tumor metastasis. Through western blotting, we verified that *TM4SF1* also regulated *DDR1* and the phosphorylation of its downstream targets *AKT, ERK,* and *mTOR* (Fig. 4e). To further identify whether TM4SF1 regulated DDR1/AKT/mTOR pathway, mTOR inhibitor Dactolisib was used, Knocking down of TM4SF1 can further down-regulated DDR1/AKT pathway after inhibitor treatment. (Fig. 4d) These results suggested that, through *DDR1*, *TM4SF1* regulates the *AKT/ERK* pathway and is involved in NSCLC chemo-resistance.

## Discussion

*TM4SF1* is an integral membrane glycoprotein that can transport extracellular signals to the cytoplasm. Originally identified as a tumor-specific antigen, *TM4SF1* has been reported to be over-expressed in many epithelial cancers, such as pancreatic cancer [7], prostate cancer [17], liver cancer [5], and especially lung cancer [18]. Additionally, *TM4SF1* has been reported to be an oncogene promoting tumor cell invasion metastasis, tumor angiogenesis, and the progression of multiple cancers [15].

Chemo-resistance is the key mechanism of the unfavorable outcome of lung cancer. Limited efficacy, significant toxicity, and the poor initial performance status of chemo-therapy was reported in advanced lung cancer [19] .Finding a biomarker that cloud be an early predict marker that can predict lung cancer patients’ response to chemo-therapy is in urgent need.

In the present study, we examined the function of TM4SF1 in NSCLC, particularly its role in NSCLC chemo-resistance. First, we examined *TM4SF1* expression in NSCLC cell lines and paired lung cancer tissue and adjacent non-cancerous tissue, by using RT-PCR and semi-quantitative PCR, respectively. *TM4SF1* was up-regulated in both lung cancer cell lines and tissues, thus suggesting that it may be a potential oncogene in NSCLC. Then, through analysis of online databases, we found that *TM4SF1* is related to important clinicopathological features of NSCLC. Online data suggested its high expression was found to be associated with the older people, and smokers. In addition, high expression of *TM4SF1* was associated with poor patient survival and outcome. Thus, the expression of *TM4SF1* may be a potential biomarker for early determination of lung cancer patient outcomes and treatment responses. Through in vitro cell function experiments, we verified that the silencing of *TM4SF1* with siRNA inhibited proliferation of the NSCLC cell lines A549 and H1299, as well as invasion and migration, thus confirming that *TM4SF1* has an oncogenic function in NSCLC. Interestingly, through MTS, we found that *TM4SF1* knockdown enhanced the chemo-sensitivity of NSCLC cell lines to cisplatin and paclitaxel. Cisplatin and paclitaxel are the most commonly used and key reagents in NSCLC chemo-therapy. Cisplatin is known as a cell cycle-non-specific chemo-therapy drug, whereas paclitaxel induces G2/M arrest. TM4SF1 promoted chemo-resistance to both reagents in NSCLC, thus suggesting that it regulates cancer chemo-resistance through inducing cell cycle arrest and other underlying mechanisms beyond cell cycle regulation, such as inducing cell apoptosis resistance and interacting with a signaling pathway strongly involved in tumor chemo-resistance. To demonstrate the above hypothesis regarding the mechanism by which *TM4SF1* regulates NSCLC chemo-sensitivity, we used flow cytometry and found that *TM4SF1* silencing in A549 and H1299 cell lines induced apoptosis and cell cycle arrest at the G2/M phase. This result verified that inducing tumor resistance to apoptosis and regulating the cell cycle are the mechanisms underlying the promotion by *TM4SF1* of NSCLC chemo-resistance. Secondly, we explored the possible signaling pathway involved in this chemo-resistance effect by using western blotting. DDR1 belongs to the large family of receptor tyrosine kinases based on the presence of a catalytic kinase domain with a distinct extracellular Discoidin (DS) homology domain [20]. TM4SF1 has been reported to couple with the collagen receptor tyrosine kinase DDR1 in breast cancer progression [9] and pancreatic cancer invasion [10]. DDR1 in turn interacts with MAPKs [16, 21] and is the up-stream regulator of the AKT pathway [11, 22]. Both pathways have been reported to interact with each other [23] and to be highly involved in tumor chemo-resistance [24]. With MAPKs and is an up-stream regulator of the AKT pathway. Therefore, by interacting with DDR1, TM4SF1 may regulate key genes in both the MAPK and AKT pathways, thus inducing NSCLC chemo-resistance. As verified by western blotting, *TM4SF1* knockdown resulted in the down-regulation of DDR1 and consequently inhibited the phosphorylation of Akt and ERK. mTOR is involved in both pathways, and its phosphorylation was also disrupted. Thus, through regulating the DDR1/ERK/Akt-mTOR pathway, TM4SF1 induces NSCLC chemo-resistance. TM4SF1 may therefore serve as a potential biomarker for predicting the treatment response in lung cancer chemo-therapy.

In general, most previous studies have focused on the role of TM4SF1 in regulating tumor invasion and metastasis. The present study demonstrated the multiple functions of TM4SF1 in NSCLC, especially its regulation of chemo-resistance and its role in regulating DDR1/ERK/Akt-mTOR pathway. These results provide another direction for the study of TM4SF1 in cancer development and demonstrate the potential value of TM4SF1 in predicting lung cancer chemo-response.

## Conclusions

Our findings suggest that *TM4SF1* is an oncogene in NSCLC. Overexpression of *TM4SF1* is a common event in NSCLC. The expression of *TM4SF1* was closely related to patient age, smoking habits and poor survival time,so detecting the expression level of *TM4SF1* may be a potential approach in the early determination of prognosis and treatment response of NSCLC. Through in vitro cell function analyze, we demonstrated that the silencing of *TM4SF1* inhibited NSCLC cell proliferation, invasion, migration and especially enhanced lung cancer cells chemo-sensitivity to cisplatin and paclitaxel. Interestingly, by western blot, we verified that interacting with *DDR1* and its downstream target ERK/Akt-mTOR is the possible mechanism of *TM4SF1* enhanced NSCLC chemo-sensitivity*.*

However, more patients and a longer follow-up time are needed to verify the correlation between *TM4SF1* expression and patient survival, treatment response and other clinical aspects. This will provide more detailed information of the role of *TM4SF1* in NSCLC early diagnosis, precision classification and treatment. Furthermore, in the exploration of the molecular mechanism underlying the oncogenic function of *TM4SF1*, more studies will be needed to find more direct evidences that *TM4SF1* directly regulates *DDR1* and might be involved in the network of *DDR1* signaling pathways.

## Abbreviations

Akt: Protein kinase B (PKB)
DDR1: discoidin domain receptor 1
ERK: Extracellular signal-regulated kinase
MAPK: Mitogen-activated protein kinase
mRNA: Messenger Ribonucleic Acid
mTOR: Mechanistic target of rapamycin
MTS: (3-(4,5dimethylthiazol-2-yl)-5-(3-carboxymethoxyphenyl)-2-(4-sulfophenyl)-2H-tetrazolium)
NSCLC: Non-small cell lung cancer
PARP: Poly ADP-ribose polymerase
PI3K: Phosphoinositide 3-kinase
TM4SF1: Transmembrane-4 L-six family member-1
